# Downregulation of Genes Involved in Metabolism and Oxidative Stress in the Peripheral Leukocytes of Huntington's Disease Patients

**DOI:** 10.1371/journal.pone.0046492

**Published:** 2012-09-27

**Authors:** Kuo-Hsuan Chang, Yi-Chun Chen, Yih-Ru Wu, Wan-Fen Lee, Chiung-Mei Chen

**Affiliations:** Department of Neurology, Chang Gung Memorial Hospital, Chang Gung University College of Medicine, Taipei, Taiwan; University of Florida, United States of America

## Abstract

**Background:**

Huntington's disease (HD) is caused by expanded CAG repeats encoding a polyglutamine tract in the huntingtin (HTT) protein. A number of differentially-expressed protein molecules have been identified in striatum of HD animal models. Here we examined if the expression changes could be visualized in the peripheral leukocytes of HD patients and pre-symptomatic HD (PreHD) carriers.

**Methods and findings:**

The expression levels of 17 candidate genes that differentially expressed in striatum between transgenic HD and wild-type mice in literature were measured in the peripheral leukocytes of 4 PreHD carriers, 16 HD patients and 20 healthy controls. Four genes majorly involved in metabolism and oxidative stress response, including *AHCY1*, *ACO2*, *OXCT1* and *CAP1*, demonstrated consistent downregulation in peripheral leukocytes of both PreHD carriers and HD patients, while *UCP2* was only down-regulated in HD patients.

**Conclusion:**

These results provide potential peripheral biomarkers to indicate disease onset in preclinical stage, and to monitor the efficacy of early treatment. Further studies of a large series of preHD carriers and symptomatic HD patients will be warranted to verify the findings and examine if these markers correlate with clinical features.

## Introduction

Huntington's disease (HD), characterized by choreiform movements, cognitive impairment and psychiatric symptoms, is an autosomal-dominant neurodegenerative disorder [1]. The major pathological presentation of HD is a regional degeneration of neurons in the striatum and cortex, which leads to movement disorders and dementia [2]. The genetic cause of HD is a long polyglutamine tract encoded by an expanded CAG trinucleotide repeats in the exon 1 of *Huntingtin* (*HTT*) [1]. The normal *HTT* gene has 35 or fewer repeats in the N-terminal region, whereas the appearance of neurological symptoms is associated with 36 or more CAG repeats in the *HTT* gene [1]. This polyglutamine expansion leads to a conformational change in the HTT and subsequently causes intracellular aggregates and neuronal degeneration [2], [3].

While extensive therapeutic strategies were developed, none of them proved to be effective in halting the disease progression. One of the major drawbacks of the clinical trial in treatments is lack of a useful biomarker that can be used as the primary end-point to test the efficacy. Thus a sensitive and practical biomarker is an unmet need for the validation of new therapeutic strategies. Given that HTT is expressed ubiquitously, and a central nervous system sample from HD patients is practically difficult to access, a biomarker in peripheral tissue, especially from blood, should be more feasible as an indicator for the disease status. Although the pathology of HD is mainly in the striatum, a few studies have identified substantial biochemical deficits in peripheral tissues [4], [5], [6], [7], [8], [9], [10], [11]. These peripheral changes include increased stress-induced lymphoblast apoptosis and vacuolation [9], [10], upregulation of H2A histone family, member (H2AFY) in leukocytes [6], increased A(2A) receptor binding sites in blood platelets [8], pro-catabolic serum metabolite profiles [11], increased IL-6 and clusterin in plasma [5], reduced 24S-hydroxycholesterol in plasma [7], and increased oxidative stress and mitochondrial dysfunction in peripheral blood [4]. Furthermore, parallel peripheral and neuronal dysfunctions have been shown [12], [13], [14], [15], [16]. Increased oxidative damage to DNA was found in the urine, plasma and striatal microdialysates of HD mice [13]. Lower Akt activation status [14], reduced cAMP and ATP/ADP ratio [15], abnormal immune activation [12], and reduced creatinine kinase-BB have been demonstrated in both peripheral blood and brain of HD [16].

Transcriptional dysregulation is one of mandatory pathogenic mechanisms in HD [17], [18]. Differential expression profiles in human peripheral tissues using oligonucleotide microarrays have been reported, whereas these two reports showed inconsistent results [19], [20]. Recently, one study has also shown that the similar disruption pattern of RNA transcription in the brain could be visualized in peripheral blood [21]. Studies have shown a number of proteins that were up- or down-regulated specifically in the striatum of transgenic HD mouse models [22], [23]. Since these results provide candidate molecules as potential biomarkers and the mRNA expression in peripheral blood may parallel the striatal changes in HD, we thus set out to examine if the expression of these molecules are altered in peripheral leukocytes of preHD and HD patients.

## Results

To gain new insights into the pathological mechanisms of neuronal degeneration in HD patients and identify potential biomarkers, we chose 17 candidate genes that expressed differentially in striatum between transgenic HD and wild-type mice in literature (Table 1) [22], [23]. The expression levels of these genes were measured on human leukocytes of HD patients. Due to its paucity of gene expression in human leukocytes [24], [25], [26], *UCP1* was replaced by its homologue *UCP2*. Amongst 14 genes down-regulated in the striatum of HD mice, 5 genes, including *AHCY*, *ACO2*, *OXCT1*, *UCP2*, *CAP1*, were in good agreement with the results in previous animal studies (Table 1). By contrast, all genes up-regulated in the striatum of HD mice displayed the similar expression preferences in HD patients.

**Table 1:** Summarized RT-PCR validation results of the 17 genes in human leukocytes.

Gene ID	Gene name	Protein name	Regulation in mouse straitum (HD vs wildtype)	Fold change in human leukocyte (HD vs control)	*P* value*
**191**	**AHCY**	**S-adenosyl-L-homocysteine hydrolase**	**Down ** [**22**]	**0.53**	**<0.001**
**50**	**ACO2**	**Aconitase 2**	**Down** [22]	**0.36**	**0.001**
**5019**	**OXCT1**	**3-oxoacid CoA transferase 1**	**Down** [22]	**0.72**	**0.001**
**7351**	**UCP2**	**Uncoupling protein 2**	**Down (UCP1)** [23]	**0.81**	**0.003**
**10482**	**CAP1**	**Adenylyl cyclase-associated protein 1**	**Down** [22]	**0.69**	**0.007**
5579	PRKCB	Protein kinase Cβ	Down [22]	0.86	0.056
5138	PDE2A	Phosphodiesterase 2A	Down [22]	1.26	0.17
5530	PPP3CA	Protein phosphatase 3, catalytic subunit, alpha isozyme	Down [22]	0.90	0.20
2280	FKBP1A	FK506 binding protein 1A	Down [22]	0.86	0.21
22933	SIRT2	Sirtuin-2	Down [22]	0.91	0.31
2752	GLUL	Glutamate-ammonia ligase	Down [22]	0.91	0.43
3098	HK1	Hexokinase-1	Down [22]	0.96	0.77
7534	YWHAZ	Tyrosine 3-monooxygenase/tryptophan 5-monooxygenase activation protein, zeta polypeptide	Down [22]	0.99	0.88
5110	PCMT1	Protein-L-isoaspartate(D-aspartate) O-methyltransferase	Down [22]	0.99	0.91
1738	DLD	Dihydrolipoamide dehydrogenase	Up [22]	0.83	0.95
25824	PRDX5	Peroxiredoxin 5 precursor	Up [22]	1.28	0.11
3421	IDH3G	Isocitrate dehydrogenase 3 (NAD+) γ	Up [22]	1.05	0.33

* Comparison of the gene expression levels of HD patients (n = 16) with controls (n = 20).

Since the expression levels of *AHCY*, *ACO2*, *OXCT1*, *UCP2* and *CAP1* were lower in the striatum of HD mouse models and the human leukocytes when compared with their wild-type and the controls respectively, they serve as potential biomarkers to indicate the disease stage or progression not only for animal studies but also for clinical trials. To test this hypothesis, we compared the expression levels of peripheral leukocytes between HD patients, preHD carriers and controls. The results showed that the mRNA expression levels of *AHCY* (0.0064±0.00067 vs 0.0074±0.00076 vs 0.0121±0.00137, *P* = 0.003), *ACO2* (0.20±0.014 vs 0.20±0.034 vs 0.56±0.087, *P*<0.001), *OXCT1* (0.0043±0.00037 vs 0.0036±0.00029 vs 0.0059±0.00054, *P* = 0.039) and *CAP1* (0.094±0.0095 vs 0.059±0.0052 vs 0.135±0.0109, *P* = 0.004) were significantly reduced in both HD patients and preHD carriers when compared with the controls by ANCOVA with adjustment of age and gender (Table 2). *UCP2* mRNA expression level of leukocyte was significantly reduced in HD patients (0.047±0.0018, *P* = 0.011) compared with preHD carriers (0.065±0.0070) and the controls (0.058±0.0029). With the estimated standard deviations in each marker, at the level of 0.05, present sample sizes achieve a power of 100%, 76%, 88%, 93%, and 99.5% for AHCY, OXCT1, UCP, CAP, and ACO2, respectively, to detect differences in the mean of each marker between case and control groups. To understand the effect of medications and severity, we calculated the correlation between gene expression levels and age, medications, or UHDRS by covariate-adjusted generalized linear model. All factors did not present significant correlation with gene expression levels (data not shown).

**Table 2:** Clinical characteristics and gene expression levels of the symptomatic HD patients, PreHD carriers and the controls.

Parameter	HD patients (n = 16)	PreHD carriers (n = 4)	Controls (n = 20)	Fold change (HD vs control)	Fold change (PreHD vs control)	*P* value‡
Gender (female/male)	5/11	1/3	6/14			
Age (years)	42.19±2.34	26.50±2.02#	39.85±2.22			
Age at symptom onset (years)	38.43±2.47					
Disease duration (years)	3.50±0.64					
Expanded CAG repeat No	45.69±1.45	42.25±0.25				
UHDRS						
Motor score	24.31±5.16	0				
Independence scale	84.38±4.83	100				
Functional capacity	10.31±0.85	13				
Drugs						
Dopamine antagonist (%)	8/16	0	0			
SSRI	5/16	0	0			
Amantadine	5/16	0	0			
Gene expression						
AHCY	0.0064±0.00067*	0.0074±0.00076*	0.0121±0.00137,	0.53	0.61	0.003
ACO2	0.20±0.014*	0.20±0.034*	0.56±0.087,	0.36	0.36	<0.001
OXCT1	0.0043±0.00037*	0.0036±0.00029*	0.0059±0.00054,	0.72	0.61	0.039
UCP2	0.047±0.0018**	0.065±0.0070	0.058±0.0029	0.81	1.12	0.011
CAP1	0.094±0.0095*	0.059±0.0052*	0.135±0.0109,	0.69	0.44	0.004

HD: Huntington's Disease. SSRI: selective serotonin uptake inhibitor. UHDRS: The Unified Huntington's Disease Rating Scale. Scale ranges (normal to most severe) include motor score (0 to 124), independence score (100 to 10), and functional capacity (13 to 0).

‡ : *P* value of ANCOVA with adjustment of age and gender.

* : Statistically significant in comparison with controls, *P*<0.05, ANCOVA with *post-hoc* Bonferroni test;

** : Statistically significant in comparison with PreHD and controls respectively, *P*<0.05, ANCOVA with *post-hoc* Bonferroni test;

# : Statistically significant in comparison with HD patients and controls respectively, *P*<0.05, ANOVA.

## Discussion

The application of a biomarker identified in animal models lies in the reproducibility in human samples. Although a number of animal studies have shown a long list of candidate biomarkers in HD mice or flies, the reappearance of these results in human system is uncertain. Here we validated that the expression changes of candidate biomarkers obtained from the striatum of HD mouse models are also present in human peripheral leukocytes of HD patients and preHD carriers. Of 17 genes quantified by RT-PCR, 5 down-regulated genes (*AHCY*, *ACO2*, *OXCT1*, *UCP2*, *CAP1*) were in good agreement with the results of animal studies.

The *AHCY* gene encodes S-adenosylhomocysteine hydrolase, which catalyzes the hydrolysis of S-adenosylhomocysteine (SAH) to adenosine and homocysteine [27]. It is known that SAH, strongly inhibits many S-adenosylmethionine (SAM)-dependent methyltransferases [27] that is crucial for the DNA, RNA and histone methylations. Therefore, S-adenosylhomocysteine hydrolase is important for maintaining the proper methylation potential in the cell. Moreover, SAH hydrolysis is the only source of homocysteine in mammals [28]. Thus reduced *AHCY* expression would obviously affect a wide variety of cellular processes. *AHCY* knockout mice have generally confirmed this by demonstrating early embryo lethality [29]. The patients with *AHCY* mutation showed white-matter atrophy and delayed myelination [27], further suggesting the downregulation of *AHCY* in HD patients may contribute to the CNS degeneration. Considering the downregulation of *AHCY* may result in the accumulation of its upstream substrates generated from methionine, restricted methionine intake could be a logical approach for the improvement of functional deterioration in HD patients and preHD carriers.

Aconitase 2 encoded by *ACO2* converts citrate to isocitrate in the tricarboxylic acid cycle and is involved in the ATP generation [30]. Decreased aconitase activity was also found previously in the striatum of R6/2 HD mice at late stage and postmortem brains of HD patients [30], [31], [32]. Aconitase 2 has been shown to be susceptible to increased oxidative stress that would lead to inactivation of aconitase 2 activity [30]. Since mitochondrial abnormalities and increased oxidative stress have long been suggested to play an important role in neurodegeneration of HD, the decreased peripheral leukocytes *ACO2* expression level in preHD carriers could be an early peripheral event for HD.

*OXCT1* encodes a mitochondrial matrix enzyme that functions as a homodimer to catalyze acetoacetate activation to acetoacetyl-CoA, playing a critical role in ketone body utilization [33]. Ketone bodies make an important contribution to brain energy production and biosynthetic processes [34]. In addition, several observations support the notion that ketone bodies exert neuroprotective effects [34]. The implication of OXCT1 in generation of ketone bodies, both in brain and peripheral tissues, suggests its important role in energy deficit in HD. Several lines of evidence have suggested metabolic deficits in HD (see review in Naia et al. [35]). Reduced ATP production has been shown in brain of presymptomatic and symptomatic HD mice [15], [36]. In preHD carriers and symptomatic HD patients, there is strong evidence of hypometabolism in the brain, especially in the basal ganglia [37], [38], [39]. A systemic metabolic defect associated with early weight loss has been noted in HD patients as well [40]. Our study for the first time demonstrated down-regulated *OXCT1* in preHD carriers and HD patients, suggesting an impairment of proper energetic supply from ketone bodies in HD. Supplement of ketogenic diet may be beneficial for the symptomatic improvement in HD patients.

The mitochondrial protein called uncoupling protein 2 encoded by *UCP2* plays an important role in generating heat and burning calories by creating a pathway that allows dissipation of the proton electrochemical gradient across the inner mitochondrial membrane [41]. *UCP2* is widely expressed in adult human tissues, including brain and macrophages [41]. In addition to the generation of ATP, *UCP2* has been identified as an inducible neuroprotective protein by decreasing the concentrations of reactive oxygen species inside mitochondria [42]. In the mouse overexpressing human *UCP2*, brain damage was diminished after experimental stroke and traumatic brain injury, and neurologic recovery was enhanced [43]. In cultured cortical neurons, *UCP2* overexpression reduced cell death and inhibited caspase-3 activation induced by oxygen and glucose deprivation [43]. In this study, the downregulation of *UCP2* is specific in HD patients, but not seen in PreHD patients, suggesting its potential role in association with the disease progression. Given that increased oxidative stress, impaired metabolism, mitochondrial dysfunction, and their interplay contribute to neuronal dysfunction in HD, the downregulation of *UCP2* in the peripheral leukocytes may be used as a potential biomarker to monitor the disease deterioration and the treatment response.

*CAP1* encodes a multifunctional protein with several structural domains involved in actin binding, adenylate cyclase association, and oligomerization [44]. Upon treatment with a number of agents that induce apoptosis, CAP1 rapidly translocates to the mitochondria [45]. This translocation is a proapoptotic event because CAP-knockdown cells are resistant to induction of apoptosis [45]. Therefore we proposed that the downregulation of CAP1 in peripheral leukocytes may have a protective effect through anti-apoptosis.

Systemic gene or protein expression analyses have been proven to provide useful candidate biomarkers, which may indicate disease status and are important for testing potential therapeutic strategies [46]. Although combining data sets obtained from independent research groups may identity biomarkers in a more reliable manner, a huge discrepancy existed amongst the studies of HD peripheral blood using microarray. For example, the global gene expression in lymphocytes from HD patients failed to show similar changes that were observed in freshly isolated blood samples [19], [20]. Although some of the HD-related changes in the expression of 12 genes identified by Borovecki et al. were confirmed in one independent study [47], the levels of serum mRNA markers (*ROCK1* and *ANXA1*) were unaffected by HD in another study [48]. The study of gene expression in peripheral lymphocytes by Runne and colleagues also failed to show consistent results found in brains of HD patients [19]. Similarly, the available studies in literature did not uncover the potential biomarkers revealed in our study. These conflicts may be originated from the differences in sample preparations, disease status, technologies, and individual biological status. Thus standard methods will be necessary to appropriately attenuate the variability in confounding factors in such studies. Furthermore, it will be promising to combine genomic and proteomic approaches with neuroimaging to successfully detect biomarkers related to disease progression in HD. Nevertheless, the down-regulated genes identified in this study highlight that treatments aimed at correcting metabolic deficits and decreasing oxidative damage may be beneficial to HD patients. In addition, these molecules may serve as the potential good indicators for evaluating efficacy of preclinical and clinical treatments aimed at improving metabolic dysfunction and oxidative stress. Given that the numbers of our patients and preHD carriers are small, a larger, multi-center, longitudinal study regarding the correlation of these gene expressions with clinical and neuroimaging features will determine the role of their applications as biomarkers for HD, and might shed light on the development of novel treatments.

## Materials and Methods

### Ethics statement

This study was performed under a protocol approved by the Institutional Review Boards of Chang Gung Memorial Hospital and all examinations were performed after obtaining written informed consents.

### Study population

Twenty subjects with HD, including 4 preHD carriers and 16 symptomatic HD patients, and 20 healthy controls were recruited in this study. Unified Huntington's Disease Rating Scale (UHDRS) were recorded for each patient [49]. The scale ranges (normal to most severe) of UHDRS include total motor score (0 to 124), independence score (100 to 10), and total functional capacity (13 to 0). None of the patients or the controls had systemic infection, autoimmune diseases, malignancies, or chronic renal, cardiac, or liver dysfunction.

### Sample collection

Blood samples were collected into EDTA-containing tubes from HD patients, PreHD carriers, and the controls after obtaining informed consent. The blood was collected into PaxgeneTM blood RNA tube (Pre-AnalytiX, Qiagen, Valencia, CA). Total RNA of leukocytes was extracted using the PaxgeneTM blood RNA Extraction Kit (Pre-AnalytiX, Qiagen, Valencia, CA), and transferred into the RNeasy MinElute spin column (RNeasy MinElute Cleanup Kit, Qiagen, Valencia, CA) for RNA purification and concentration. RNA quality was determined using the A260/A280 absorption ratio.

### Real-time polymerase chain reaction (RT-PCR)

Total RNAs were collected from the peripheral blood leukocytes of HD subjects and controls. RNA was converted to cDNA using the SuperScript III First-Strand (Invitrogen). PCR results were generated using the 5′-nuclease assay (TaqMan) if the probe is commercially available and the ABI 7900HT Sequence Detection System (Applied Biosystems). Primers for *ACO2* designed by using Primer Express software, Version 2.0, Applied Biosystems were used for amplification of Aco2 cDNA. Primers were designed to amplify transcripts across an exon junction to avoid genomic DNA contamination. Each reaction included cDNA from 100 ng of RNA, 900 nM of each primer and 100 nM of probes or primers and Universal PCR Master Mix (Applied Biosystems). Probe sequence information is indicated in Table 3. PCR parameters were 50°C for 2 min, 95°C for 10 min, 40 cycles of 95°C for 15 sec, 60°C for 1 min. Each sample was assessed in duplicate. Cycle threshold (CT, the fractional cycle number where the fluorescent signal reaches detection threshold) in each reaction was set in the linear range. Relative expression values were normalized to *β-ACTIN*. Relative gene expressions were calculated using the 2^ΔCT^ method, ΔCT = CT (*β-ACTIN*) – CT (target gene).The CTs of *β-ACTIN* across different samples ranged between 20 and 22. For each set of values, data were expressed as means ± standard error (SE). Differences between groups were evaluated by Student's *t*-test, ANOVA, or ANCOVA (adjusted by age and gender) with *post-hoc* Bonferroni test where appropriate. Correlations of UHDRS (motor scale, independence scale and functional capacity), size of expanded polyglutamine or disease duration with levels of mRNA were analyzed by covariate-adjusted generalized linear model (adjusted by age and gender). All *P*-values were two-tailed. The values of *P*<0.05 were considered significant.

**Table 3:** Lists of assay ID and probe sequence for RT-PCR.

Gene	Assay ID	Probe sequence
AHCY	Hs00426322_m1	CTACAAAGTCGCCGACATCGGCCTG
ACO2		Forward: GCCGTCACTCAGGAGTTTGGReverse: CGTAGTTCTCGTCTCCGATCAC
OXCT1	Hs00166467_m1	GACACCACAGGGCACACTTGCAGAG
UCP2	Hs01075225_m1	GCTCTGAGCATGCCAGCATTGGGAG
CAP1	Hs02860542_g1	GTCAAAGTTCAGGTAATGGGTAAAG
PRKCB	Hs00176998_m1	GGCAGAAATTTGAGAGGGCCAAGAT
PDE2A	Hs01042255_m1	GTCATGGAGAGGCACCACTTTGCTC
PPP3CA	Hs00174223_m1	AGATGGATTTGATGGTGCAACAGCT
FKBP1A	Hs00356621_g1	GCACTACACCGGGATGCTTGAAGAT
SIRT2	Hs00247263_m1	CAGAGCGAACGCTGTCGCAGAGTCA
GLUL	Hs00374213_m1	TGTGGCTGGGAACACCTTCCACCAT
HK1	Hs00175976_m1	GCACCCACAGTATTCCCGGCGTTTC
YWHAZ	Hs01122445_g1	ACAAGAAAGGGATTGTCGATCAGTC
PCMT1	Hs00193600_m1	TGTACCCCAGGCGCTAATAGATCAG
DLD	Hs01022655_m1	ATTCTTGGACCAGGTGCTGGAGAAA
PRDX5	Hs00201536_m1	CCCCAATCAAGGTGGGAGATGCCAT
IDH3G	Hs00188065-m1	GCGTGGCCCTGAAGGGCAACATCGA
